# Social factors associated with trust in the health system in northern Sweden: a cross-sectional study

**DOI:** 10.1186/s12889-022-13332-4

**Published:** 2022-05-04

**Authors:** Mazen Baroudi, Isabel Goicolea, Anna-Karin Hurtig, Miguel San-Sebastian

**Affiliations:** Department of Epidemiology and Global Health, Umeå University, 901 87 Umeå, Sweden

**Keywords:** Trust, Health system, Inequalities, Sweden

## Abstract

**Background:**

Despite the importance of having trust in the health system, there is a paucity of research in this field in Sweden. The aim of this study was to estimate the level of trust in the health system and to assess the factors associated with it in northern Sweden.

**Methods:**

A cross-sectional survey was conducted in 2014 in the four northern regions of Sweden. A total of 24 795 participants (48% response rate) aged 18 to 84 years were involved in the study. A log-binomial regression was used to measure the association between sociodemographic factors and trust in the health system.

**Results:**

Two thirds of the participants (68.5%) reported high trust in the health system i.e. had very much or quite a lot confidence in the health system. Women had lower prevalence of trust compared to men (PR = 0.96; 95% CI = 0.94–0.98) while older participants had a higher trust compared to youth (PR = 1.11; 95% CI = 1.06–1.16). Participants with lower level of education, those who experienced economic stress, those who were born outside Sweden and those living in small municipalities also had lower prevalence of trust in the health system. Conversely, lower income was associated with higher trust (PR = 1.08; 95% CI = 1.04–1.12). Finally, a strong relationship between higher social capital (having emotional and instrumental support, horizontal trust, and higher social participation) and trust in the health system was also found.

**Conclusions:**

Trust in the health system was moderately high in northern Sweden and strongly associated with sociodemographic and social capital factors. Trust is a complex phenomenon and a deeper exploration of the relation between trust in the health system and sociodemographic factors is needed.

## Background

Trust in the health system is considered one of the most important and yet least researched aspects of a well-functioning health system [1]. Research has shown that the lack of trust in health institutions can be associated with underutilisation of healthcare and delayed help-seeking behaviour [2], poor adherence to treatment [3], increased psychological distress, mental disorders and poor self-rated health [4].

Even though the meaning of trust in the health system can be debatable and lacks a precise definition [5], the common ground of trust is the optimistic acceptance of vulnerability and anticipation that the trustee will act to the best interests of the trustor [6]. Contrary to satisfaction, which is mainly built on previous experiences, trust additionally reflects expected individuals’ behaviours, and therefore has been proposed as a potential indicator of health services performance [7]. Furthermore, it has been argued that trust in healthcare as an institution can help build values and moral fibres in the society, and thus create more trust in the state [8].

Trust in the health system is not equally distributed in the population. Lack of trust has been linked in the literature to several sociodemographic characteristics such as being a man, of younger age, and with low levels of education and income [9, 10]. Social capital including horizontal trust, i.e. trust in other people, has also been discussed as a strong determinant of trust in healthcare providers [9]. In addition, the effects of racialisation on trust have received a large share of attention, especially in the US, where black people tend to have lower trust in the health system [11].

Research on trust in the health system in the Swedish context is limited. A study from 2007 in southern Sweden showed that 73.3% of the participants had very or rather high trust in the health system [4]. Other studies have shown that lower levels of trust in the Swedish health system were associated with being a man and of younger age. Additionally, the Sámi Indigenous population and immigrants have also reported less trust in the health system [12–14].

Traditionally, the Swedish welfare system is focused on universal health care, equality and personal autonomy [15]. However, variations between regions in Sweden exist, due partly to the decentralised health system. In northern Sweden, a scattered population in large areas, long distances from healthcare facilities and the difficulty of recruiting and retaining healthcare workers add further complexity to the organisation of the healthcare system. Furthermore, rurality tends to increase the sense of exclusion and contribute to the marginalisation of healthcare in these areas [16]. All these factors might contribute to decrease trust in healthcare by increasing the feeling of isolation and the reluctance to seek healthcare [17].

There is a need for a better understanding of the social factors influencing trust in health system in order to develop targeted interventions to increase trust [1]. This is especially of importance in the context of the rural and sparsely populated areas, such as those living in the northernmost regions.

The aim of this study was to explore the level of trust in the health system and to assess sociodemographic and social capital factors associated with it in northern Sweden.

## Methods

### Setting and population

The Health on Equal Terms (HET) is a cross-sectional survey that aim to follow people’s health and lifestyle over time including physical and mental health, healthcare visits, dental health, drug consumption, work environment, security and social relations. The survey is conducted every four years in the four northernmost regions of Sweden (Norrbotten, Västerbotten, Västernorrland and Jämtland). These regions contain less than one tenth of the Swedish population but more than half the area of the country. The population density is as low as 4 inhabitants per square kilometre, compared to approximately 50 in the rest of Sweden [18].

We used HET 2014 since the most recent HET 2018 questionnaire lacks the outcome of our interest, i.e. trust in the health system. Using the Swedish population register as a sampling frame, Statistics Sweden (SCB) sent the questionnaire randomly to a representative sample of 16 to 84 year-old participants. A total of 24 795 participants were included in this study (48% participation rate). The questionnaire includes inquiries about participants’ health, social capital and economic and demographic factors, besides the level of trust in the health system. The dataset was linked with register-based individual data including education level, occupation, income, civil status and place of birth.

### Measures

#### Trust

The level of trust in the health system was measured through the question: “How much confidence do you have in the health system?”, with five options: very much, quite a lot, not very much, none at all and have no opinion. For the purpose of our study, the variable was categorised into: high trust, corresponding to ‘very much’ and ‘quite a lot’ (coded as 1); and low trust, including ‘not very much’ and ‘none at all’ (coded as 0); participants who did not respond to the question or answered ‘have no opinion’ (6.5% and 3% respectively) were considered as missing.

The inclusion of the social factors in the models was guided by the literature; those social factors that had shown a potential association with trust in the literature [9–14, 19], and were available in the dataset, were included.

#### Sociodemographic factors

*Age* was categorised into four groups: 16 to 25, 26 to 45, 46 to 65 and 66 to 84 years-old. *Sex* was self-reported and divided into men and women. *Education* was classified into low (less than three years of secondary education), medium (up to two years of post-secondary education) and high (three years of post-secondary education or more). *Occupation* was classified into white (skilled) and blue (unskilled) collar workers based on the Swedish Socioeconomic Classification (SEI-1982). *Annual disposable individual income,* which covers all personal income after the deduction of taxes and debts, was categorised into five quintiles; the poorest coded as 1 and the richest coded as 5. *Economic stress* was assessed by asking if the participant had ever had difficulties in managing regular expenses (e.g. for food, rent, bills) in the past twelve months. *Birth place* was dichotomised into Sweden and outside the country. *Municipality size was* categorised into municipalities with more than 50 000 inhabitants; municipalities with 10 000 to 50 000 inhabitants with a hospital; 10 000 to 50 000 inhabitants without a hospital; and municipalities with less than 10 000 inhabitants. *Civil status* was divided into married/cohabitating; not-cohabitating including divorced and widowed.

#### Social capital

We used four of the most commonly used individual level indicators to measure social capital [20]. *Horizontal trust* was captured by the question: “Do you think that people generally can rely on other people?”. *Social participation* was assessed by the question: “Have you taken part in any of the following activities in the last 12 months?”. This included for instance, study circles, meetings, art, sport and recreational events, religious gatherings and demonstrations. The response was categorised into three groups based on the number of activities the participants took part in: no activity; some but not many activities (1 to 4) or many activities (5 or more). *Instrumental support* was measured by the question “Can you get help from any person or persons if you have practical problems or are ill? For instance, get advice, borrow things, and help with shopping or repairs”. *Emotional support* was measured by the question “Do you have anyone you can share your innermost feelings with and confide in?”. The last two questions were binary, allowing a yes or no answer.

#### Statistical methods

The different sociodemographic and social capital characteristics of the sample were calculated as percentages together with the frequency of trust in the health system for each variable. The association between trust in the health system and the diverse independent factors was summarised with the prevalence ratio using log-binomial regression. Model 1 included the unadjusted values, while model 2 was adjusted to the socioeconomic factors. In model 3, social capital variables were added to the statistically significant variables in model 2.

Regression models were tested for multi-collinearity with the variance inflation factor. Sample weights were tested but no different results were observed, and therefore unweighted data were used. Analysis were stratified by sex to test for possible interactions but did not show different results. Prevalence ratios and 95% confidence intervals were reported considering significant level to be a *p*-value of less than 0.05. Stata 15 software was used for the analysis.

#### Ethical consideration

The data were collected by the northern regions of Sweden where informed consents to use the data for research purposes were obtained from all the participants. Ethical approval to use HET data in the current study was obtained from the regional ethical review board in Umeå (No: 2015/134-31Ö).

## Results

Table 1 shows the sample characteristics with the proportion of participants with high trust in the health system for each variable. Participants had roughly equal distribution in the sex and occupation subgroups. About half of the participants had low education level and almost 40% were living in municipalities with less than 10 000 inhabitants. Most of the participants were born in Sweden (93.6%), married or co-habiting (70.5%), had no economic stress (89.2%) and good social capital (79.7% had horizontal trust and 95.5% instrumental support).

**Table 1:** Sample characteristics and proportion of people with high trust in the health system in each subgroup, “Health in equal terms” survey, Northern Sweden 2014

	2014 (N: 24 795)	
	n (%)	Trust%
**total**	24 795 (100)	68.5
**Age**		
16–25 yrs	2 348 (9.9)	63.7
26–45 yrs	6 260 (26.4)	63.1
46–65 yrs	7 331 (31.0)	68.9
66–84 yrs	7 744 (32.7)	74.4
**Sex**		
Man	11 328 (46.3)	69.5
Woman	13 145 (53.7)	67.6
**Education**		
high	4 137 (17.0)	75.0
Medium	8 179 (33.6)	65.7
low	12 051 (49.5)	68.0
**Occupation**		
White-collar	12 056 (49.8)	71.2
Blue-collar	12 135 (50.2)	65.6
**Income**		
Quintile 5 (richest)	4 945 (20.0)	69.5
Quintile 4	4 945 (20.0)	69.4
Quintile 3	4 947 (20.0)	68.1
Quintile 2	4 946 (20.0)	68.0
Quintile 1	4 946 (20.0)	67.3
**Economic stress**		
No	21 965 (89.2)	70.1
Yes	2 671 (10.8)	55.3
**Birth place**		
Sweden	23 198 (93.6)	69.0
Outside Sweden	1 597 (6.4)	61.3
**Municipality size**		
> 50 000 inhibitants	6 004 (24.2)	72.4
10 to 50 with hospital	4 797 (19.4)	68.9
10 to 50 without hospital	4 260 (17.2)	68.2
< 10 000 inhibitants	9 734 (39.3)	66.0
**Civil status**		
Married/cohabiting	17 483 (70.5)	69.2
not-cohabiting	7 312 (29.5)	66.8
**Horizontal trust**		
No	4 895 (20.3)	50.6
Yes	19 176 (79.7)	73.2
**Social participation**		
no activities	3 969 (16.0)	62.6
1 to 4 activities/yr	15 482 (62.4)	68.0
5 to 12 activities/yr	5 344 (21.6)	73.6
**Instrumental support**		
Cannot get help	1 087 (4.5)	50.3
Can get help	23 073 (95.5)	69.5
**Emotional support**		
No	2 581 (10.8)	58.6
Yes	21 527 (89.3)	69.8

Around two thirds of the participants (68.5%) reported high trust in the health system. This proportion was similar among men and women or by civil status. Around three quarters of senior citizens (74.4%) and those with high education (75.0%) reported high trust in the health system. The variation in the level of trust in the health system was small by income quintiles, though greater variations in the economic stress and occupation variables were observed; only 55.3% of those who suffered economic stress and 65.6% of the blue-collar workers reported high trust in the health system. Similarly, around two thirds of participants born outside Sweden (61.3%) and of those living in small municipalities (66%) reported high trust in the health system. Additionally, the level of trust in the health system varied substantially according to social capital, with those with higher social capital (in all four dimensions) reporting higher trust (Table 1).

### Factors associated with trust in the health system

The crude analysis revealed a statistically significant association between trust in the health system in all the selected variables, with income being statistically significant only in the poorest quintile (Table 2, model 1). In model two, all explanatory factors continued to be statistically significant except civil status.

**Table 2:** Crude and adjusted prevalence ratio showing the effects of sociodemographic and social capital factors on the trust in the health system, “Health in equal terms” survey, Northern Sweden 2014

	Model 1	Model 2	Model 3
	Crude PR (95%CI)	Adjusted PR (95%CI)	Adjusted PR (95%CI)
**Age**			
16–25 yrs	1	1	1
26–45 yrs	0.99 (0.95–1.03)	0.97 (0.93–1.01)	**0.95 (0.91–0.99)**
46–65 yrs	**1.08 (1.04–1.12)**	**1.05 (1.01–1.10)**	1.03 (0.98–1.07)
66–84 yrs	**1.17 (1.13–1.21)**	**1.14 (1.09–1.19)**	**1.11 (1.06–1.16)**
**Sex**			
Man	1	1	1
Woman	**0.97 (0.96–0.99)**	**0.97 (0.95–0.99)**	**0.96 (0.94–0.98)**
**Education**			
High	1	1	1
Medium	**0.88 (0.85–0.90)**	**0.90 (0.87–0.92)**	**0.90 (0.89–0.93)**
Low	**0.91 (0.89–0.93)**	**0.89 (0.87–0.91)**	**0.91 (0.89–0.94)**
**Occupation**			
White-collar	1	1	
Blue-collar	**0.92 (0.90–0.94)**	**0.97 (0.95–0.99)**	0.99 (0.97–1.01)
**Income**			
Quintile 5 (richest)	1	1	1
Quintile 4	1.00 (0.97–1.02)	**1.03 (1.00–1.06)**	**1.04 (1.01–1.07)**
Quintile 3	0.98 (0.95–1.01)	1.01 (0.99–1.04)	**1.03 (1.00–1.06)**
Quintile 2	0.98 (0.95–1.01)	1.01 (0.98–1.04)	**1.04 (1.01–1.07)**
Quintile 1	**0.97 (0.94–0.99)**	**1.04 (1.01–1.08)**	**1.08 (1.04–1.12)**
	1		
**Economic stress**			
No	1	1	1
Yes	**0.79 (0.76–0.82)**	**0.83 (0.80–0.86)**	**0.88 (0.85–0.92)**
**Birth place**			
Sweden	1	1	1
Outside Sweden	**0.89 (0.85–0.93)**	**0.90 (0.86–0.95)**	**0.94 (0.90–0.98)**
**Municipality size**			
> 50 000 inhibitants	1	1	1
10 to 50 with hospital	**0.95 (0.93–0.98)**	**0.96 (0.93–0.98)**	**0.96 (0.94–0.99)**
10 to 50 without hospital	**0.94 (0.92–0.97)**	**0.95 (0.92–0.97)**	**0.96 (0.93–0.99)**
< 10 000 inhibitants	**0.91 (0.89–0.93)**	**0.93 (0.91–0.95)**	**0.93 (0.91–0.95)**
**Civil status**			
Married/cohabiting	1	1	
not-cohabiting	**0.97 (0.95–0.98)**	0.98 (0.96–1.00)	
**Horizontal trust**			
No	1	-	1
Yes	**1.45 (1.40–1.49)**	-	**1.36 (1.32–1.41)**
**Social participation**			
no activities	1	-	1
1 to 4 activities/yr	**1.09 (1.06–1.12)**	-	**1.05 (1.02–1.08)**
5 to 12 activities/yr	**1.17 (1.14–1.21)**	-	**1.12 (1.08–1.16)**
**Instrumental support**			
Cannot get help	1	-	1
Can get help	**1.38 (1.30–1.47)**	-	**1.18 (1.11–1.26)**
**Emotional support**			
No	1	-	1
Yes	**1.19 (1.15–1.23)**	-	**1.05 (1.02–1.09)**

Model 2 adjusted for sex, age, education level, occupation, income, economic stress, birth place, municipality size and civil status; Model 3 adjusted for all the above beside the level of horizontal trust, social participation, instrumental support and emotional support. Statistically significant results in bold

After controlling for social capital factors in model 3, all the sociodemographic factors except occupation continued to be statistically significant. The analysis revealed that older people reported a higher prevalence of trust compared to the youngest group (PR = 1.11; 95% CI = 1.06–1.16), while women had lower prevalence of trust in the health system than men (PR = 0.96; 95% CI = 0.94–0.98). Regarding socioeconomic status, low level of education was associated with a lower prevalence of trust (PR = 0.91; 95% CI = 0.89–0.94), while lower income was related with higher trust (PR = 1.08; 95% CI = 1.04–1.12). Having economic stress (PR = 0.88; 95% CI = 0.85–0.92), being born outside Sweden (PR = 0.94; 95% CI = 0.90–0.98) or living in small municipalities (PR = 0.93; 95% CI = 0.91–0.95) were associated with lower trust in the health system. (Table 2, model 3).

All social capital factors were associated with trust in the health system (Table 2, model 3). Having horizontal trust was associated with a 36% higher prevalence of trust (PR = 1.36; 95% CI = 1.32–1.41). Similarly, high social participation (PR = 1.12; 95% CI = 1.08–1.16), existing instrumental (PR = 1.18; 95% CI = 1.11–1.26) and emotional support (PR = 1.05; 95% CI = 1.02–1.09) were also significantly associated to higher trust.

## Discussion

Around two thirds of the participants (68.5%) had high trust in the health system. Younger participants, women, those born outside Sweden, living in smaller municipalities, having lower education, experiencing economic stress or having lower social capital were associated with a lower trust in the health system. However, lower income was associated with higher trust.

The study showed a moderately high level of trust in the health system in northern Sweden. This can be compared to the national average of 61% reported in 2019 and to the 73.3% reported in southern Sweden in 2007 [4, 21]. An international comparison of 31 countries using the same question to assess trust in the health system was conducted between 2011 and 2013 and showed that Belgium had the highest level with 72% reporting high trust, followed by Spain and Scandinavian countries, with percentages ranging from 56 to 59%. Large differences between the countries were reported: Germany (42%), France and the United Kingdom (around 30%) and the US (19%) [19]. Comparing countries is however a challenge due to the differences in health systems and cultures and to the lack of a standard method to measure trust [22].

The relation between age and trust is in line with previous research, which has shown that older populations tend to have more trust in the health system [12, 23, 24]. This might be explained by the modernisation theory, which postulates that the economic, political and cultural changes in the post-industrial societies result in a rejection of traditional social institutions. Younger generations are supposed to have greater shifts in cultural values, which in turn might lead to greater mistrust in the institutions; that is, these structural changes make it harder for young people to aspire to the same things to which the previous generations had aspired [25].

This study observed lower trust among women compared to men. Even though these differences were small, this finding was surprising since an earlier study in 2009 from northern Sweden showed an opposite relationship [12], and the literature often shows lower trust among men [9]. Women are usually more exposed to health services through their own experiences or through accompanying their spouse or children. This higher exposure to services together with the continuous threat of closing down healthcare services, including maternity units, in the rural parts of northern Sweden [26], might have led to different experiences and perceptions of healthcare among women, and consequently to lower trust in the health system [27].

According to our results, lower education and experiencing economic stress were also associated with lower trust, while lower income was associated with higher trust. The literature points in different directions regarding the relation between trust and income and education. Some studies have shown a relation between low income and lower trust in healthcare providers and health information [9, 10], while other studies have observed that higher education and income were associated with lower trust in the health system [19, 23, 24]. The different direction of the association between economic stress and annual disposable individual income and trust in this study suggests that these two variables are capturing distinct aspects of socioeconomic status which need further exploration.

Participants born outside Sweden reported lower trust in the health system compared to those born in Sweden. Earlier research in Sweden indicated similarly low trust in Swedish healthcare among refugees and immigrants [13, 14]. These differences could be explained by the perceived discrimination of the system towards participants born outside Sweden [11]. The different health system expectations of immigrants could further lead to worse experiences and thus to lower trust in the health system [13, 14].

According to our results, participants living in smaller rural municipalities had lower trust in the health system compared to those living in larger urban ones. However, a multinational study with 31 countries showed opposite results; people living in urban areas showed lower trust in the health system [19]. Results have been observed in China that are similar to the case of northern Sweden, while no differences between urban and rural areas were reported in the UK [23, 28]. Our results are probably context specific to northern Sweden where there are smaller rural municipalities with no hospitals and relatively long distances from healthcare facilities, and where rural citizens experiencing a policy of abandonment by central authorities. For example, closing some services in rural areas led to a mistrust in institutions in general and in healthcare in particular [17].

Lastly, our study showed that those with better social capital had more trust in the health system. A complex and bidirectional relation between horizontal trust and social or public trust in the health system has been discussed in the literature [29]. Studies have shown that social capital may improve trust in, quality of and access to the health system by altering users’ perception of healthcare [9]. This reinforces the social capital theory, indicating that social experiences and engagement in social activities have an important role in building social and horizontal trust, which in turn helps to build trustworthy organisations [9].

### Strengths and limitations

HET survey offers a unique set of data that contains several sociodemographic variables, the trust in health system variable and a representative large sample of the region. Moreover, supplementing the HET with register-based sociodemographic data decreased reporting bias, especially for sensitive data such as income and place of birth.

Several issues should however be considered when interpreting the findings. First, while the 48% response rate is comparable to other national surveys, we cannot exclude selection bias since the composition of the population in the regions included indicates, for example, higher proportion of people born outside Sweden (9.1% compared to 6.4% who answered this survey). The extent and direction of such bias could not be assessed. Second, given the study design, a reverse causality cannot be excluded. For example, horizontal trust has been associated with high trust in the health system, while trust in government and social institutions might reinforce horizontal trust [30]. Third, some relevant factors such as healthcare needs, experiences in healthcare, health care workers’ behaviours, continuity of care and availability of services could not be measured in this study. Since these factors are important in understanding trust, their inclusion might have altered some of our findings. Additionally, since ethnicity was not measured in the survey, we were not able to assess the Sámi Indigenous population’s trust in the health system. Finally, our results are time and place bounded. It is unclear whether our findings can be generalised to other settings in or outside of Sweden. While the current Covid-19 pandemic might have affected the level of trust in health system in northern Sweden, we would however expect that the pattern of trust among people across the different social characteristics to remain similar.

## Conclusions

Our study shows a moderately high trust in the health system in northern Sweden and a strong relation between social capital and trust in the health system. Other factors such as sex, age, education, income, economic stress, place of birth and municipality size were also related to trust in the health system, to different extents. Trust in the health system is a complex contextual issue and a deeper understanding of the relationship between trust in the health system and sociodemographic factors is needed to be able to reduce these inequalities. Targeting rural areas, migrants and people with lower socioeconomic status with interventions that increase social capital could be promoted as a way, among others, to increase trust in the health system.

## Data Availability

The data that support the findings of this study are available from the register holders, the respective regions, but restrictions apply to the availability of these data, which were used under license for the current study, and so are not publicly available. Data are however available from the authors upon reasonable request and with permission of Norrbotten, Västerbotten, Västernorrland and Jämtland regions.

## Abbreviations

HET: Health on Equal Terms survey
PR: Prevalence ration
SCB: Statistics Sweden
